# Supplementary material for: Controllable light capsules employing modified Bessel-Gauss beams
Source: Sci Rep. 2016 Jul 8;6:29001. doi: 10.1038/srep29001 (PMC4937432; doi:10.1038/srep29001)
Supplement: Supplementary Information [file srep29001-s1.pdf]

Controllable light capsules employing modified Bessel-Gauss beams

Lei Gong,[1] Weiwei Liu,[1] Qian Zhao,[1] Yuxuan Ren,[2] Xingze Qiu,[1] Mincheng Zhong[1] & Yinmei Li[1,*]

[1]*Department Department of Optics and Optical Engineering, University of Science and Technology of China, Hefei, 230026, China*

[2]*National Center for Protein Sciences Shanghai, Institute of Biochemistry and Cell Biology, Shanghai Institutes for Biological Sciences, CAS, Shanghai, 201210, China*

**Corresponding author: liyinmei@ustc.edu.cn*

Supplementary Information

Video Legends:

Movie 1: One-dimensional transverse intensity profile of zero-order modified Bessel-Gauss beam along with the increasing parameter σ ($\sigma = r_{d0}/\omega_0$), which is displayed in the yellow frame in real time.

Movie 2: The longitudinal intensity distribution of zero-order modified Bessel-Gauss beam along with the increasing parameter σ.

Movie 3: Optical manipulation of the trapped core-shell magnetic microparticles in the vertical direction. The red circles indicate the range of the light capsules, and the arrows respresent the directions of the flowing fluid. Because of the soft boundary of the light capsule, the particle can partially go beyond it caused by the viscous drag force of the fluid. Besides, defocus blur can be observed in this process because the lower confinement in the axial direction induced by the asymmetric bottle shape. Scale bar, 5μm.

Movie 4: Optical manipulation of the trapped core-shell magnetic microparticles in the horizontal direction. The red circles indicate the range of the light capsules, and the arrows respresent the directions of the flowing fluid. Scale bar, 5μm.
